# The impact of tooth loss on the risk of cognitive impairment among community-dwelling older adults: a prospective cohort study

**DOI:** 10.1186/s12889-026-27244-0

**Published:** 2026-04-11

**Authors:** Yu-Kai Lin, Wan-Yu Lin

**Affiliations:** Department of Health and Welfare, College of City Management, University of Taipei, No.101, Sec.2, Zhongcheng Rd., Shilin Dist, Taipei City, 111036 Taiwan

**Keywords:** Oral health, Early detection, Cognitive impairment, Health examination, Prospective cohort study

## Abstract

**Background:**

Oral health problems, such as tooth loss, are common among older adults and have been linked to chronic diseases and functional decline. However, evidence based on rigorous longitudinal designs and large-scale population databases remains limited. This study aimed to investigate the association between tooth loss and the risk of cognitive impairment among community-dwelling older adults.

**Methods:**

This prospective cohort study included adults aged 65 years and older who participated in a health examination program in Taipei between 2013 and 2022. After applying inclusion and exclusion criteria, 8,260 participants were included in the final analysis and followed until the onset of cognitive impairment, their last health examination, or the end of the study period. Tooth loss was assessed through professional dental examinations, and cognitive impairment was evaluated using the Ascertain Dementia 8-Item Questionnaire (AD-8). Cox proportional hazards models were used to estimate hazard ratios (HRs) and 95% confidence intervals (CIs).

**Results:**

Older adults with 9–16 missing teeth had a significantly higher risk (HR = 1.28; 95% CI: 1.06–1.55) of developing cognitive impairment. Associations for more severe tooth loss (≥ 17 missing teeth) were not statistically significant, possibly due to smaller subgroup sizes and compensatory factors such as denture use. Higher risks were also observed among male participants, those aged 65–84 years, those who were married, had lower educational attainment, or did not engage in regular exercise.

**Conclusion:**

Tooth loss is a prevalent condition among older adults and may contribute to an increased risk of cognitive impairment. These findings underscore the importance of regular oral health assessments and targeted preventive strategies as part of comprehensive geriatric care to support cognitive health in aging populations.

**Clinical trial number:**

Not applicable.

**Supplementary Information:**

The online version contains supplementary material available at 10.1186/s12889-026-27244-0.

## Background

As the global population ages, Alzheimer’s disease and other forms of dementia have become leading causes of death and disability among older adults [1]. In addition, the rising costs of medical and healthcare services have significantly affected the quality of life and social functioning of older individuals [2, 3]. Previous study have identified five key domains of risk factors for cognitive decline and dementia: genetic predisposition, unhealthy lifestyle habits, multiple chronic conditions, abnormal physiological markers, and psychological or psychiatric disorders [4]. Given the irreversible nature of cognitive deterioration, the development of preventive strategies and early detection methods is both crucial and urgent [5].

Among these risk domains, lifestyle modification represents one of the most feasible approaches for older adults, particularly in their daily lives. These modifiable behaviors include maintaining physical activity, adopting a healthy diet, quitting smoking, avoiding excessive alcohol consumption, and managing blood pressure, cholesterol, and blood glucose levels, as well as engaging in social interactions [3]. Although many studies have examined the relationship between healthy lifestyles and the risk of cognitive impairment, dementia, and Alzheimer’s disease [6–8], evidence regarding the role of dietary habits remains inconsistent. One potential explanation for this inconsistency is the presence of oral health problems, such as tooth loss, which may influence both nutritional intake and cognitive function.

Tooth loss has been linked to a range of adverse health outcomes, including hypertension, diabetes mellitus, frailty, functional disability, and reduced quality of life [9–13]. Moreover, tooth loss has been associated with increased mortality due to cancer and cardiovascular diseases [14]. In recent years, several studies have also explored the potential association between tooth loss and cognitive impairment. For example, Galindo-Moreno et al. analyzed two national health surveys from the United States and reported that the number of remaining teeth was associated with cognitive decline. However, due to limitations in the survey structure, cognitive status was assessed using only a single item related to memory and confusion, without comprehensive cognitive evaluation [15]. Similarly, using a community cohort in China, Yang et al. found that older adults with 20 or more natural teeth had a lower risk of cognitive impairment. Nonetheless, their study relied on self-reported dental status rather than clinical dental examinations [16].

To address these limitations and further clarify the role of tooth loss as a modifiable lifestyle factor in the prevention of cognitive decline, this study aims to investigate the association between tooth loss and cognitive impairment using a prospective cohort design. Furthermore, we explore the moderating effects of demographic and socioeconomic factors on this relationship.

## Methods

### Study population and selection criteria

This study utilized data from the Taipei Older Adults Health Examination Database, covering the years 2012 to 2022. The database enrolled community-dwelling residents of Taipei City, targeting indigenous adults aged 55 years and older and non-indigenous adults aged 65 years and above. In total, 33,774 participants were enrolled in 2013, which served as the baseline year for this study. To ensure that participants were in relatively stable health conditions, we reviewed health indicators from both 2012 (the year prior to baseline) and 2013 to assess health consistency or variability.

**Fig. 1:**
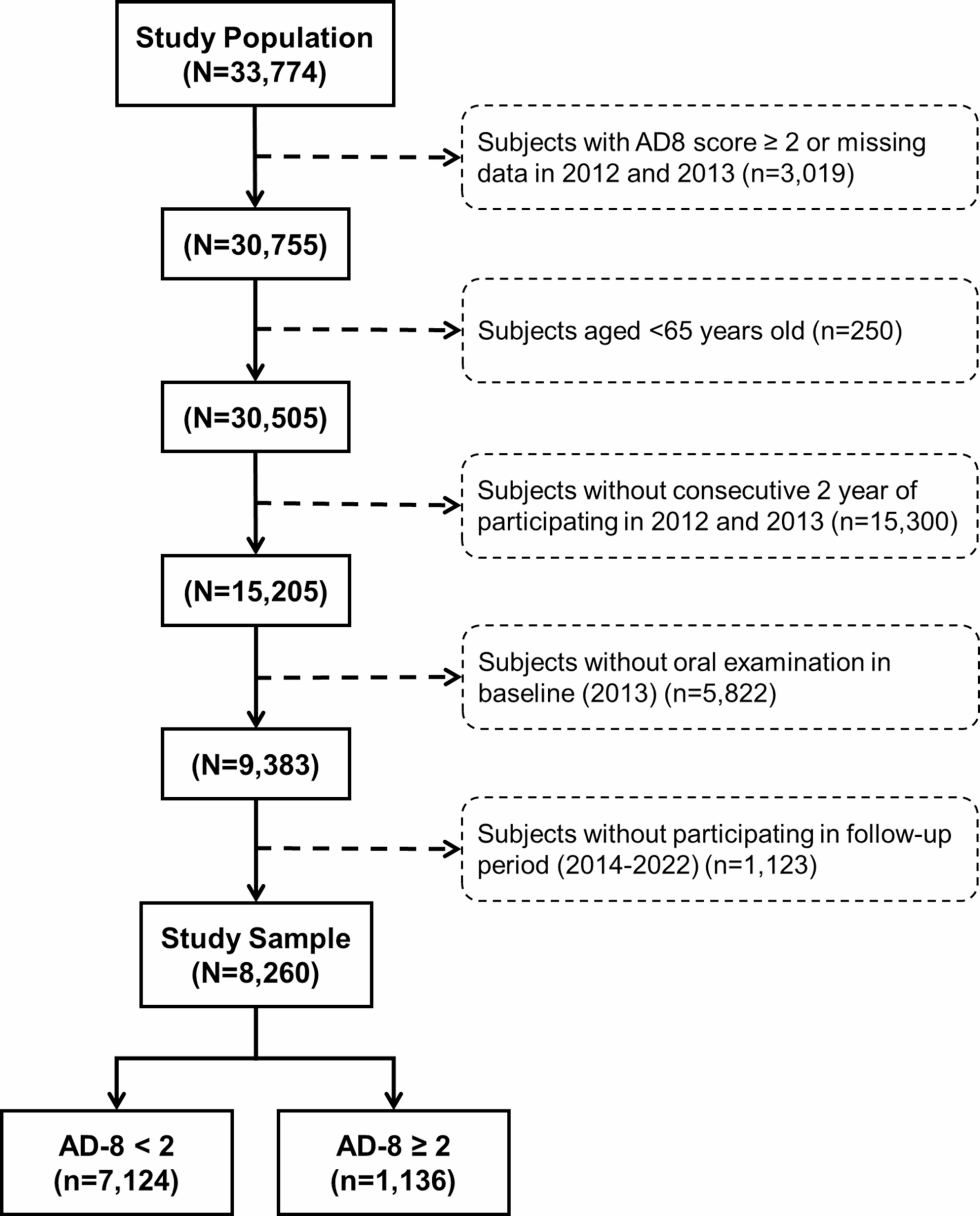
Study Flowchart

Participants were excluded from the analysis if they had an Ascertain Dementia 8-Item Questionnaire (AD-8) score of ≥ 2 or were missing data in either 2012 or 2013 (*n* = 3,019), were younger than 65 years at baseline (*n* = 250), did not participate in both 2012 and 2013 (*n* = 15,300), lacked oral examination data in the baseline year (*n* = 5,822), or did not participate in any follow-up examinations between 2014 and 2022 (*n* = 1,123). After applying these exclusion criteria, a total of 8,260 participants were included in the final analysis (study flowchart shown in Fig. 1). The study protocol was reviewed and approved by the Institutional Review Board of the University of Taipei (IRB-2021-002), and all procedures adhered to the Declaration of Helsinki and relevant ethical standards.

### Independent variable: number of tooth loss

Tooth loss was assessed by licensed dentists at contracted medical institutions in Taipei City. During routine dental examinations, dentists recorded the number of missing natural teeth, as well as the presence of dental caries, retained roots, prostheses, and the condition of oral mucosa and periodontal tissues. For the purpose of this study, tooth loss was defined as the number of missing natural teeth. Teeth that were present but clinically considered non-functional-such as retained roots, teeth with severely destroyed crown, or teeth with marked mobility།were recorded separately and were not classified as missing teeth. Prosthetic teeth were also not included in the tooth loss count, thereby avoiding misclassification and double counting across oral health indicators. All oral health data were documented using standardized examination forms and subsequently entered into the research database by trained research assistants. The number of missing teeth was categorized into five groups: no tooth loss, loss of 1–8 teeth (approximately one-quarter), loss of 9–16 teeth (approximately one-half), loss of 17–24 teeth (approximately three-quarters), and loss of 25–32 teeth (nearly all teeth).

### Outcome variable: cognitive impairment

Cognitive impairment was evaluated using the Ascertain Dementia 8-Item Questionnaire (AD-8), a brief, informant-based screening tool developed to detect changes in cognitive functioning across eight domains. These domains include judgment and decision-making, engagement in hobbies and activities, repetitive speech, ability to learn new information, temporal orientation, financial management, memory for scheduled events, and overall thinking ability [17]. For example, participants were asked whether they were more easily deceived, had lost interest in previous activities, frequently repeated stories, experienced difficulty learning new things, had memory decline, encountered challenges in handling complex finances, missed important appointments, or demonstrated noticeable cognitive or memory-related changes.

The AD-8 was selected for this study because it is easy to administer, requires minimal administration time, and is well suited for large-scale community-based screening among older adults. Unlike performance-based cognitive assessments such as the Mini-Mental State Examination (MMSE), Montreal Cognitive Assessment (MoCA), and Clinical Dementia Rating (CDR), the AD-8 is an informant-based questionnaire and is less influenced by participants’ educational attainment and cultural background [18]. In addition, the AD-8 has demonstrated good sensitivity for detecting early cognitive changes and has been widely used and validated in Taiwan and other Asian populations [19, 20]. Previous study has also shown good reliability and validity of the AD-8, with strong correlations with MMSE, MoCA, and CDR scores [21].

Each item was scored as either “Yes, there is a change” (1 point) or “No, no change or unsure” (0 points), yielding a total score ranging from 0 to 8. A score of 2 or more was used to define cognitive impairment. Previous validation study has reported acceptable diagnostic performance of the AD-8, with a sensitivity of 74%, specificity of 86%, and an area under the curve (AUC) of 0.83 [17]. In this study, trained interviewers administered the AD-8 through one-on-one interviews using standardized procedures to ensure data quality and consistency.

### Covariates

Demographic, socioeconomic, medical, physiological, and lifestyle-related variables were included as covariates in this study. Sex was self-reported as male or female, and age was calculated by subtracting the year of birth from the baseline year. Marital status was recorded as either married or unmarried, and educational attainment was grouped into three levels: more than 13 years, between 7 and 12 years, and 6 years or fewer. Solitary living status was documented as living alone and not.

Medical history was assessed through self-reported physician diagnoses of hypertension, heart disease, and diabetes mellitus. Physiological data, including fasting glucose (AC), total cholesterol (TCHO), triglycerides (TG), high-density lipoprotein (HDL), and low-density lipoprotein (LDL), were obtained via venous blood sampling. Systolic and diastolic blood pressure, body weight, and height were measured by trained nursing staff using standardized equipment, and body mass index (BMI) was calculated as weight in kilograms divided by height in meters squared.

Lifestyle-related variables included smoking habits, alcohol consumption, and exercise levels. Smoking status was categorized as never, occasional, or daily; for analytical purposes, occasional and daily smokers combined. Alcohol use was classified as non-drinker, occasional drinker, or frequent drinker. Exercise was assessed based on self-reported weekly duration and categorized as none, less than 150 min per week, or 150 min or more. Missing responses for any covariates were coded as missing data.

### Statistical analysis

Descriptive statistics were used to compare baseline characteristics between participants with and without cognitive impairment, defined as AD-8 scores < 2 and ≥ 2, respectively. Continuous variables were analyzed using Student’s t-tests, while categorical variables were compared using Chi-square tests. Cox proportional hazards regression models were applied to examine the association between tooth loss and incident cognitive impairment, with hazard ratios (HRs) and 95% confidence intervals (CIs) estimated.

A stepwise modeling approach was adopted. The unadjusted model (Model 1) included only the independent variable. Model 2 adjusted for age and sex, while Model 3 further included marital status and education. Model 4 additionally adjusted for exercise habits, and Model 5 was the fully adjusted model incorporating demographic, socioeconomic, medical, physiological, and lifestyle covariates that were statistically significant in baseline group comparisons.

To evaluate the robustness of the primary findings, sensitivity analyses were conducted using alternative definitions of tooth loss based on previous literature. First, following the classification proposed by Yang et al. [16], tooth loss was reclassified by converting the number of missing teeth to the corresponding number of remaining natural teeth, assuming a full dentition of 32 teeth, and categorized into four groups: ≥20, 10–19, 1–9, and 0 remaining teeth. Second, based on review by Gotfredsen et al. [22], which have identified the retention of 20 natural teeth as a clinically relevant threshold for adequate masticatory efficiency and ability, tooth loss was dichotomized using 20 remaining teeth as the cutoff point. These alternative categorizations were applied to the fully adjusted Cox proportional hazards models.

Stratified analyses were conducted to assess potential effect modification by sex, age group (65–74, 75–84, ≥ 85 years), marital status, education level, and exercise habit. A two-tailed p-value < 0.05 was considered statistically significant. All statistical analyses were performed using Stata version 14.

## Results

Table 1 presents the baseline characteristics of the study sample, in which 1,136 participants (13.75%) developed cognitive impairment by the end of the follow-up period. Older adults with AD-8 scores ≥ 2 had an average of 6.21 missing teeth, which was higher than the average of 5.37 among those with AD-8 scores < 2. The mean age of participants with cognitive impairment was 77.03 years, also significantly older than those without cognitive impairment. In the AD-8 ≥ 2 group, a higher proportion of individuals were unmarried (22.10%) and had an educational attainment of 6 years or less (29.58%) compared to those in the AD-8 < 2 group. Additionally, the prevalence of heart disease and diabetes was significantly higher among participants with AD-8 scores ≥ 2. Notably, the proportion of individuals without a regular exercise habit was greater in the cognitively impaired group than in their counterparts without impairment.

**Table 1:** Baseline characteristics of study participants by cognitive impairment status (*N* = 8,260)

	AD8 < 2	AD8 ≥ 2	*P*-value	Model 1 (Crude model) HR (95% CI)
	*n* = 7,124 (86.25%)	*n* = 1,136 (13.75%)		
	*n (% in column) / Mean ± SD*			
Tooth Loss (number)	5.37 ± 6.55	6.21 ± 7.17	< 0.01^†^	1.02 (1.01–1.03)^†^
Tooth Loss (group)				
No tooth loss	1,897 (26.63)	291 (25.62)	< 0.01^†^	Ref
Loss of 1–8 teeth	3,842 (53.93)	558 (49.12)		0.91 (0.79–1.05)
Loss of 9–16 teeth	850 (11.93)	176 (15.49)		1.32 (1.10–1.60)^†^
Loss of 17–24 teeth	324 (4.55)	64 (5.63)		1.35 (1.03–1.77)^*^
Loss of 25–32 teeth	211 (2.96)	47 (4.14)		1.60 (1.18–2.18)^†^
Sex				
Female	3,437 (48.25)	589 (51.85)	< 0.05^*^	Ref
Male	3,687 (51.75)	547 (48.15)		0.90 (0.80–1.01)
Age (number)	75.21 ± 6.00	77.03 ± 6.12	< 0.01^†^	1.08 (1.07–1.09)^†^
Age (group)				
65–74 years	3,654 (51.29)	458 (40.32)	< 0.01^†^	Ref
75–84 years	2,894 (40.62)	532 (46.83)		1.76 (1.56-2.00)^†^
≥ 85 years	576 (8.09)	146 (12.85)		3.22 (2.67–3.89)^†^
Marital Status				
Married	5,838 (81.95)	885 (77.90)	< 0.01^†^	Ref
Unmarried	1,285 (18.04)	251 (22.10)		1.36 (1.18–1.56)^†^
Missing	1 (0.01)	0 (0.00)		
Education Attainment				
≥ 13 years	2,493 (34.99)	341 (30.02)	< 0.01^†^	Ref
7–12 years	2,987 (41.93)	459 (40.40)		1.19 (1.03–1.37)^*^
≤ 6 years	1,644 (23.08)	336 (29.58)		1.69 (1.45–1.96)^†^
Solitary Status				
Non-solitary	6,744 (94.67)	1,074 (94.54)	0.86	Ref
Solitary	380 (5.33)	62 (5.46)		1.07 (0.83–1.39)
Hypertension				
Without	3,817 (53.58)	611 (53.79)	0.90	Ref
With	3,307 (46.42)	525 (46.21)		1.08 (0.96–1.22)
Heart Disease				
Without	6,048 (84.90)	903 (79.49)	< 0.01^†^	Ref
With	1,076 (15.10)	233 (20.51)		1.58 (1.37–1.82)^†^
Diabetes				
Without	6,312 (88.60)	974 (85.74)	< 0.01^†^	Ref
With	812 (11.40)	162 (14.26)		1.39 (1.18–1.64)^†^
Smoking Habit				
Non-smoker	6,824 (95.79)	1,090 (95.95)	0.36	Ref
Smoker	276 (3.87)	45 (3.96)		1.04 (0.77–1.39)
Missing	24 (0.34)	1 (0.09)		
Alcohol Consumption				
Non-drinker	5,620 (78.89)	922 (81.16)	0.15	Ref
Occasional drinker	1,361 (19.10)	199 (17.52)		0.83 (0.71–0.96)^*^
Frequent drinker	117(1.64)	14 (1.23)		0.78 (0.46–1.32)
Missing	26 (0.36)	1 (0.09)		
Exercise Habit				
≧ 150 min/week	3,819 (53.61)	538 (47.36)	< 0.01^†^	Ref
< 150 min/week	2,486 (34.90)	431 (37.94)		1.26 (1.11–1.44)^†^
No	788 (11.06)	165 (14.52)		1.55 (1.30–1.85)^†^
Missing	31 (0.44)	2 (0.18)		
BMI (kg/m^2^)	23.97 ± 3.58	23.97 ± 3.18	0.99	1.00 (0.99–1.02)
SBP (mmHg)	132.31 ± 17.75	131.76 ± 17.80	0.33	1.00 (0.99-1.00)
DBP (mmHg)	73.69 ± 11.23	73.07 ± 11.50	0.09	0.99 (0.99-1.00)
AC (mg/dL)	101.99 ± 19.54	103.13 ± 19.91	0.07	1.00 (1.00-1.01)^†^
TCHO (mg/dL)	188.69 ± 32.88	186.25 ± 34.48	< 0.05^*^	0.99 (0.99–0.99)^†^
TG (mg/dL)	112.45 ± 88.25	113.90 ± 60.77	0.60	1.00 (0.99-1.00)
HDL (mg/dL)	56.27 ± 15.61	55.49 ± 15.69	0.12	0.99 (0.99–0.99)^*^
LDL (mg/dL)	110.35 ± 29.07	108.58 ± 29.90	0.06	0.99 (0.99–0.99)^†^

*SD* Standard deviation, *HR* Hazard ratio, *CI *Confidence interval, *BMI* Body mass index, *SBP* Systolic blood pressure, *DBP* Diastolic blood pressure, *AC* Fasting glucose, *TCHO* Total cholesterol, *TG* triglycerides, *HDL* High-density lipoprotein, *LDL* Low-density lipoprotein

^*^ *P* < 0.05

^†^ *P* < 0.01

**Fig. 2:**
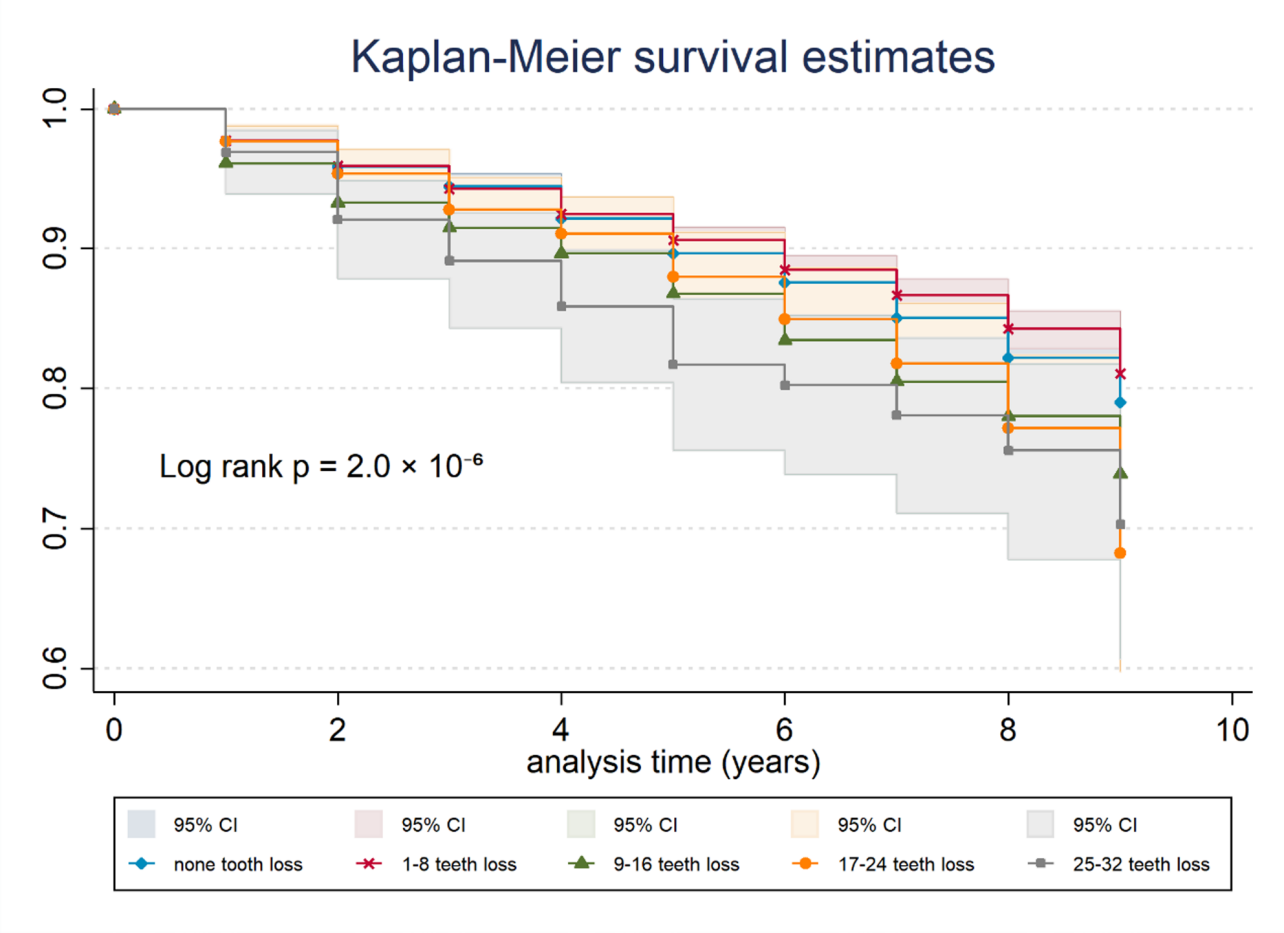
Kaplan–meier curves for cognitive impairment by tooth loss

Tables 1 and 2 present the crude and fully adjusted models examining the association between tooth loss and cognitive impairment. For brevity, the results from intermediate models (Model 2-Model 4), which progressively adjusted for age and sex, marital status and education, and exercise habits, are shown in Additional Table. Across these sequential models, the hazard ratio for participants with 9–16 missing teeth remained stable in magnitude and direction, indicating minimal attenuation after adjustment for potential confounders. In the fully adjusted model (Model 5), older adults who had lost 9–16 teeth had a 28% higher risk of cognitive impairment compared with those with no tooth loss (HR: 1.28; 95% CI: 1.06–1.55). Although participants with more extensive tooth loss (≥ 17 missing teeth) also showed elevated hazard ratios, these associations did not reach statistical significance. Kaplan-Meier survival curves further illustrated graded differences in cumulative incidence across tooth loss categories (log-rank *p* = 2.0 × 10⁻⁶; Fig. 2), with 95% confidence intervals shown for each tooth loss group.

**Table 2:** Hazard ratios for cognitive impairment by tooth loss

Tooth Loss	Model 5 (Full model)	
	HR (95% CI)	*P*-value
No tooth loss	Ref	Ref
Loss of 1–8 teeth	1.00 (0.87–1.16)	0.99
Loss of 9–16 teeth	1.28 (1.06–1.55)	< 0.05^*^
Loss of 17–24 teeth	1.18 (0.90–1.56)	0.22
Loss of 25–32 teeth	1.23 (0.90–1.68)	0.20

Full model is controlled for sex, age, marital status, education attainment, heart disease, diabetes, exercise habit, and total cholesterol

HR Hazard ratio, *CI* Confidence interval

^*^ *P* < 0.05

^†^ *P* < 0.01

To further assess the robustness of the primary findings, sensitivity analyses using alternative tooth loss categorizations were performed. As shown in Additional Table, participants with 13–22 missing teeth (corresponding to 10–19 remaining teeth) had a significantly increased risk of cognitive impairment (HR: 1.37; 95% CI: 1.13–1.66) compared with those with ≤ 12 missing teeth (corresponding to ≥ 20 remaining teeth). In contrast, participants with ≥ 23 missing teeth (corresponding to ≤ 9 remaining teeth) did not show a statistically significant increase in risk. When tooth loss was dichotomized using the 20-tooth threshold, individuals with > 12 missing teeth (corresponding to < 20 remaining teeth) had a 27% higher risk of cognitive impairment (HR: 1.27; 95% CI: 1.08–1.50).

Stratified analyses, as shown in Table 3, revealed distinct patterns across subgroups. Among older males, those with 9–16 missing teeth had a 50% increased risk (95% CI: 1.14–1.96), while those with 25–32 missing teeth had a 71% increased risk (95% CI: 1.13–2.58) of developing cognitive impairment compared to those without tooth loss. Among married individuals, those with 9–16 and 25–32 missing teeth had a 29% (95% CI: 1.04–1.60) and 43% (95% CI: 1.01–2.03) higher risk, respectively.

**Table 3:** Stratified hazard ratios for cognitive impairment by tooth loss

Tooth Loss	HR (95% CI)		
Sex	Female*n* = 4,026	Male*n* = 4,234	
No tooth loss	Ref	Ref	
Loss of 1–8 teeth	0.96 (0.79–1.16)	1.06 (0.86–1.31)	
Loss of 9–16 teeth	1.11 (0.85–1.46)	1.50 (1.14–1.96)^†^	
Loss of 17–24 teeth	1.07 (0.72–1.60)	1.32 (0.91–1.93)	
Loss of 25–32 teeth	0.86 (0.53–1.41)	1.71 (1.13–2.58)^*^	
Marital Status	Married*n* = 6,723	Unmarried*n* = 1,536	
No tooth loss	Ref	Ref	
Loss of 1–8 teeth	1.03 (0.88–1.22)	0.88 (0.65–1.18)	
Loss of 9–16 teeth	1.29 (1.04–1.60)^*^	1.25 (0.85–1.85)	
Loss of 17–24 teeth	1.18 (0.86–1.60)	1.18 (0.65–2.13)	
Loss of 25–32 teeth	1.43 (1.01–2.03)^*^	0.73 (0.35–1.51)	
Age group	65–74 years*n* = 4,112	75–84 years*n* = 3,426	≥ 85 years*n* = 722
No tooth loss	Ref	Ref	Ref
Loss of 1–8 teeth	0.97 (0.78–1.22)	1.03 (0.83–1.28)	0.94 (0.63–1.41)
Loss of 9–16 teeth	1.37 (1.00-1.86)^*^	1.25 (0.95–1.65)	1.22 (0.75–1.99)
Loss of 17–24 teeth	1.23 (0.73–2.08)	1.43 (1.01–2.02)^*^	0.61 (0.24–1.54)
Loss of 25–32 teeth	1.81 (0.97–3.36)	1.15 (0.73–1.81)	1.10 (0.59–2.06)
Education Attainment	≤ 6 years*n* = 1,980	7–12 years*n* = 3,446	≥ 13 years*n* = 2,834
No tooth loss	Ref	Ref	Ref
Loss of 1–8 teeth	1.09 (0.83–1.43)	0.99 (0.79–1.24)	0.94 (0.72–1.21)
Loss of 9–16 teeth	1.57 (1.12–2.19)^†^	1.12 (0.83–1.52)	1.24 (0.87–1.77)
Loss of 17–24 teeth	1.16 (0.71–1.90)	1.11 (0.73–1.69)	1.41 (0.84–2.38)
Loss of 25–32 teeth	1.30 (0.76–2.20)	1.26 (0.78–2.03)	1.15 (0.58–2.30)
Exercise habit	None*n* = 953	< 150 min/week*n* = 2,917	≥ 150 min/week*n* = 4,357
No tooth loss	Ref	Ref	Ref
Loss of 1–8 teeth	1.23 (0.84–1.80)	0.94 (0.75–1.18)	0.97 (0.78–1.19)
Loss of 9–16 teeth	1.29 (0.78–2.13)	1.35 (0.99–1.83)	1.23 (0.93–1.62)
Loss of 17–24 teeth	1.27 (0.59–2.75)	1.26 (0.82–1.93)	1.12 (0.75–1.66)
Loss of 25–32 teeth	2.01 (1.02–3.95)^*^	1.07 (0.60–1.91)	1.13 (0.72–1.79)

Model is controlled for sex, age, marital status, education attainment, heart disease, diabetes, exercise habit, and total cholesterol

*HR* Hazard ratio, *CI* Confidence interval

^*^ *P* < 0.05

^†^ *P* < 0.01

In terms of age stratification, participants aged 65–74 years with 9–16 missing teeth had a 37% increased risk (95% CI: 1.00–1.86), and those aged 75–84 years with 17–24 missing teeth had a 43% increased risk (95% CI: 1.01–2.02) of cognitive impairment. Among participants with an educational level of 6 years or less, those with 9–16 missing teeth had a 57% higher risk (95% CI: 1.12–2.19). Furthermore, individuals without a regular exercise habit who had lost 25–32 teeth exhibited a 101% increased risk (95% CI: 1.02–3.95) of cognitive impairment compared to those with no tooth loss.

To facilitate interpretation of these stratified results, stratum-specific sample sizes for each subgroup and tooth loss category are provided in Additional Table. Several subgroup estimates, particularly those involving severe tooth loss or the oldest age group, were based on relatively small sample sizes and should therefore be interpreted with caution.

## Discussion

This prospective cohort study, based on data from the Taipei Older Adults Health Examination Database (2013–2022), aimed to investigate the causal relationship between tooth loss and cognitive impairment among community-dwelling older adults. The findings indicate that individuals who had lost 9–16 teeth exhibited a 28% significantly higher risk of developing cognitive impairment compared to those without any tooth loss. Moreover, stratified analyses revealed that factors such as male sex, being married, having lower educational attainment, and lacking regular exercise habits moderated this association. To our knowledge, this is among the few studies employing a prospective design to explore this relationship in a large, population-based Asian cohort.

Over the nine-year follow-up period, 1,136 participants (13.75%) developed cognitive impairment. This incidence rate is comparable to those reported in similar studies. For instance, Nagatani et al. conducted a longitudinal study in Japan using data from the Kashiwa Study, which also targeted community-dwelling older adults aged ≥ 65 years. After nine years, 261 of 1,708 participants (19%) developed cognitive impairment, slightly higher than our estimate despite a similar follow-up duration and population criteria [23]. Similarly, a community-based cohort study in China reported by Zhang et al. found a 22.9% incidence of cognitive impairment over a 7-year follow-up among 8,390 older adults. They also identified education level, gender, marital status, and depression as major predictors [24]. Taken together, although the incidence of cognitive impairment varies across populations and methodologies, our findings are generally consistent with previous evidence from East Asian contexts.

In recent years, growing attention has been paid to the role of oral health—particularly tooth loss—as a modifiable risk factor for future cognitive decline. A systematic review and meta-analysis by Asher et al. synthesized findings from 47 studies and concluded that poor periodontal health and tooth loss are independently associated with increased risks of both cognitive decline and dementia [25]. However, many of the included studies were limited by short follow-up durations or suboptimal methodological quality. In contrast, Nagatani et al. employed a longitudinal design and demonstrated that older adults with oral frailty—especially those with reduced dentition—had a significantly increased risk of cognitive impairment over nine years of follow-up [23].

Similarly, a meta-analysis by Qi et al., pooling data from Europe, the Americas, and Asia, reported a 1.48-fold increased risk of cognitive impairment and a 1.28-fold increased risk of dementia among individuals with greater tooth loss [26]. In a Finnish population-based cohort with up to 15 years of follow-up, Asher et al. found that participants with more than 13 missing teeth had a 1.21-fold higher risk of lower baseline cognitive scores, a 1.30-fold greater risk of cognitive decline over 11 years, and a 1.52-fold increased risk of dementia after 15 years [27]. These findings are consistent with the present study and further support the temporal association between tooth loss and subsequent cognitive impairment. Notably, many prior studies relied on self-reported oral health measures, whereas the present study incorporated clinically assessed dental examinations, strengthening the validity of the observed associations.

An additional observation of the present study is that the association between tooth loss and cognitive impairment was most pronounced among participants with moderate tooth loss (9–16 missing teeth), whereas estimates for more severe tooth loss did not reach statistical significance. This pattern suggests that the relationship between tooth loss and cognitive impairment may reflect a non-linear or threshold relationship rather than a simple monotonic dose–response association. Moderate tooth loss may represent a critical transitional stage among individuals with partial dentition, during which masticatory efficiency and dietary intake or nutritional status begin to decline, while compensatory mechanisms—such as prosthetic rehabilitation (e.g., denture use) or dietary modification—have not yet been fully established. In contrast, individuals with more extensive tooth loss may have already adopted such compensatory strategies, which could partially mitigate the adverse impact of tooth loss on cognitive health. Supporting this interpretation, previous studies have reported that denture use is associated with a lower risk of cognitive impairment, particularly among individuals with partial tooth loss [16, 28]. Additionally, the relatively small number of participants with severe tooth loss in our cohort may have limited the statistical power to detect significant associations in this group.

Our stratified analyses suggest that the association between tooth loss and cognitive impairment may vary across several sociodemographic and lifestyle factors. In particular, males, married individuals, those with lower educational attainment, and those lacking regular exercise habits appeared to be more vulnerable. The stronger association observed among men may be partly explained by differences in health behaviors and healthcare utilization. Previous studies have reported that men generally exhibit poorer oral health behaviors and lower utilization of dental services compared with women, which may contribute to more severe periodontal disease and subsequent tooth loss [29, 30]. In addition, lifestyle-related risk factors that are more prevalent among men, such as smoking and alcohol consumption, may exacerbate systemic inflammation and vascular risk [31], both of which have been associated with cognitive decline and dementia [32]. Xu et al. reported that the rate of tooth loss between survey waves was associated with an increased risk of cognitive impairment in both men and women, suggesting that changes in oral health status may serve as an important marker of cognitive risk across sexes [33]. Furthermore, Zhao et al. reported that BMI mediated the association between tooth loss and cognitive function only among men, suggesting potential sex-specific biological mechanisms linking oral health and cognitive outcomes and partially explaining the stronger association observed among men in the present study [34].

Several biological mechanisms may underlie the observed association. First, the act of mastication stimulates trigeminal nerve input, which increases cerebral blood flow and brain activity [35, 36]. Second, as dentition deteriorates, dietary choices become restricted, often resulting in poor nutritional intake—particularly lower consumption of fruits, vegetables, protein, and complex carbohydrates—which may contribute to cognitive decline [37]. Third, periodontitis, a leading cause of tooth loss, may induce systemic inflammation through elevated levels of biomarkers such as C-reactive protein, tumor necrosis factor-α, immunoglobulin G, interleukin-1β, and interleukin-6, all of which have been implicated in neurodegenerative processes [38, 39]. Taken together, these mechanisms may be particularly relevant at a threshold stage of moderate tooth loss and may help explain why the association observed in the present study was most pronounced among participants with 9–16 missing teeth.

The primary strengths of this study lie in its large, population-based cohort drawn from a government-supported health examination program, its long-term follow-up period, and the use of clinical oral examinations conducted by licensed dentists. The outcome variable, cognitive impairment, was measured using the AD-8, a validated and widely used screening tool in aging research. Moreover, the prospective cohort design enhances the strength of causal inference between tooth loss and cognitive outcomes.

Nonetheless, several limitations should be acknowledged. First, the study population consisted of individuals who voluntarily participated in annual health check-ups, which may introduce selection bias. In addition, participants excluded due to missing baseline data or lack of oral examination information differed systematically from those included in the final analytic sample. As shown in Additional Table 4, excluded participants were more likely to be female and younger, and more frequently unmarried, with lower educational attainment and solitary living status. They also exhibited a higher prevalence of heart disease and diabetes. These differences suggest the potential for selection bias related to both sociodemographic factors and underlying health conditions. However, because several of these characteristics are associated with increased risks of cognitive impairment, the observed associations in the present study are unlikely to be overestimated and may instead represent conservative estimates.

Second, several important covariates—such as genetic predisposition, frequency of dental visits, and mental health status—were not available in the dataset and could not be adjusted for in the analyses. Genetic factors may be associated with both oral health and neurodegenerative processes through shared inflammatory or metabolic pathways, while mental health conditions, particularly depression, have been linked to poorer oral hygiene, reduced healthcare utilization, and increased risk of cognitive impairment. The absence of these variables may therefore result in residual confounding, and the direction and magnitude of the potential bias cannot be fully determined.

Third, cognitive impairment was assessed using a screening instrument rather than clinical diagnosis by specialists, which may have affected diagnostic accuracy, although the assessment tool used has been widely validated in community-based settings.

## Conclusions

In conclusion, this prospective cohort study provides evidence supporting a temporal association between tooth loss and the development of cognitive impairment among community-dwelling older adults. These findings underscore the importance of early identification and management of oral health problems as part of comprehensive geriatric care. Beyond promoting regular dental check-ups, feasible interventions may include incorporating targeted oral health screenings into routine annual geriatric assessments, particularly for older adults at higher risk of cognitive decline. In addition, public health policies that subsidize dental examinations, prosthodontic services, and denture provision for socioeconomically vulnerable or other high-risk subgroups may help reduce barriers to adequate oral care. Community-based oral health education programs focusing on preventive care and denture maintenance could further enhance oral function and overall well-being. Integrating these oral health-focused strategies into existing aging-related health promotion programs may contribute to the preservation of nutritional status, functional capacity, and cognitive health in older populations.

## Supplementary Information

Additional File 1.

## Data Availability

This study used third party data made available under license that the author does not have permission to share. Requests to access the data should be directed to Department of Health of Taipei City Government in Taiwan.

## Abbreviations

AC: Fasting glucose
AD-8: Ascertain Dementia 8-Item Questionnaire
AUC: Area under the curve
BMI: Body mass index
CDR: Clinical Dementia Rating
CIs: Confidence intervals
HDL: High-density lipoprotein
HRs: Hazard ratios
LDL: Low-density lipoprotein
MMSE: Mini-Mental State Examination
MoCA: Montreal Cognitive Assessment
TCHO: Total cholesterol
TG: Triglycerides
