# Supplementary material for: The impact of tooth loss on the risk of cognitive impairment among community-dwelling older adults: a prospective cohort study
Source: BMC Public Health. 2026 Apr 11;26:1648. doi: 10.1186/s12889-026-27244-0 (PMC13196173; doi:10.1186/s12889-026-27244-0)
Supplement: Supplementary file 1 — Additional File 1. [file 12889_2026_27244_MOESM1_ESM.docx]

**The Impact of Tooth Loss on the Risk of Cognitive Impairment among Community-Dwelling Older Adults: A Prospective Cohort Study**

**Additional Table 1. Multivariable Hazard Ratios for Cognitive Impairment Across Sequential Models**

| **Tooth Loss** | **HR (95% CI)** | | |
| --- | --- | --- | --- |
|  | **Model 2** | **Model 3** | **Model 4** |
| No tooth loss | Ref | Ref | Ref |
| Loss of 1–8 teeth | 0.98 (0.85-1.14) | 1.00 (0.87-1.15) | 1.01 (0.88-1.17) |
| Loss of 9–16 teeth | 1.27 (1.06-1.54)^✝^ | 1.27 (1.06-1.54)^✝^ | 1.29 (1.07-1.55)^✝^ |
| Loss of 17–24 teeth | 1.19 (0.91-1.56) | 1.16 (0.89-1.53) | 1.18 (0.90-1.54) |
| Loss of 25–32 teeth | 1.25 (0.92-1.70) | 1.21 (0.89-1.65) | 1.23 (0.90-1.68) |

^*^ P<0.05; ^✝^ P<0.01; Model 2 is controlled for sex and age: Model 3 is controlled for Model 2 + marital status and education attainment: Model 4 is controlled for Model 3 + exercise habit; HR: hazard ratio; CI: confidence interval

**Additional Table 2. Sensitivity Analyses Using Alternative Tooth Loss Categorizations**

**Panel A. Categorization According to Yang et al., Based on Missing Teeth**

| **Missing teeth** | **Remaining teeth** | **AD8 < 2**  **n=7,124** | **AD8 ≥ 2**  **n=1,136** | | **Model 5**  **(Full model)** |
| --- | --- | --- | --- | --- | --- |
|  |  | **n (% in column)** | | **HR (95% CI)** | |
| ≤ 12 | ≥ 20 | 6,314 (88.63) | 958 (84.33) | | Ref |
| 13-22 | 10-19 | 528 (7.41) | 123 (10.83) | | 1.37 (1.13-1.66)^✝^ |
| 23-31 | 1-9 | 223 (3.13) | 43 (3.79) | | 1.07 (0.79-1.46) |
| 32 | 0 (edentulism) | 59 (0.83) | 12 (1.06) | | 1.15 (0.65-2.04) |

^*^ P<0.05; ^✝^ P<0.01; Full model is controlled for sex, age, marital status, education attainment, heart disease, diabetes, exercise habit, and total cholesterol; HR: hazard ratio; CI: confidence interval

**Panel B. Dichotomized Classification Using the 20-Tooth Threshold (Functional Dentition)**

| **Missing teeth** | **Remaining teeth** | **AD8 < 2**  **n=7,124** | **AD8 ≥ 2**  **n=1,136** | | **Model 5**  **(Full model)** |
| --- | --- | --- | --- | --- | --- |
|  |  | **n (% in column)** | | **HR (95% CI)** | |
| ≤ 12 | ≥ 20 | 6,314 (88.63) | 958 (84.33) | | Ref |
| > 12 | < 20 | 810 (11.37) | 178 (15.67) | | 1.27 (1.08-1.50)^✝^ |

^*^ P<0.05; ^✝^ P<0.01; Full model is controlled for sex, age, marital status, education attainment, heart disease, diabetes, exercise habit, and total cholesterol; HR: hazard ratio; CI: confidence interval

**Additional Table 3. Stratum-Specific Sample sizes by Tooth Loss Category**

| **Tooth Loss** | **n (% in column)** | | | |
| --- | --- | --- | --- | --- |
| **Sex** | **Female**  **n=4,026** | | **Male**  **n=4,234** | |
| No tooth loss | 1,080 (26.83) | | 1,108 (26.17) | |
| Loss of 1–8 teeth | 2,184 (54.25) | | 2,216 (52.34) | |
| Loss of 9–16 teeth | 469 (11.65) | | 557 (13.16) | |
| Loss of 17–24 teeth | 168 (4.17) | | 220 (5.20) | |
| Loss of 25–32 teeth | 125 (3.10) | | 133 (3.14) | |
| **Marital Status** | **Married**  **n=6,723** | | **Unmarried**  **n=1,536** | |
| No tooth loss | 1,745 (25.96) | | 442 (28.78) | |
| Loss of 1–8 teeth | 3,629 (53.98) | | 771 (50.20) | |
| Loss of 9–16 teeth | 831 (12.36) | | 195 (12.70) | |
| Loss of 17–24 teeth | 324 (4.82) | | 64 (4.17) | |
| Loss of 25–32 teeth | 194 (2.89) | | 64 (4.17) | |
| **Age group** | **65-74 years**  **n=4,112** | **75-84 years**  **n=3,426** | | **≥ 85 years**  **n=722** |
| No tooth loss | 1,049 (25.51) | 903 (26.36) | | 236 (32.69) |
| Loss of 1–8 teeth | 2,446 (59.48) | 1,673 (48.83) | | 281 (38.92) |
| Loss of 9–16 teeth | 435 (10.58) | 493 (14.39) | | 98 (13.57) |
| Loss of 17–24 teeth | 122 (2.97) | 217 (6.33) | | 49 (6.79) |
| Loss of 25–32 teeth | 60 (1.46) | 140 (4.09) | | 58 (8.03) |
| **Education Attainment** | **≤ 6 years**  **n=1,980** | **7-12 years**  **n=3,446** | | **≥ 13 years**  **n=2,834** |
| No tooth loss | 563 (28.43) | 904 (26.23) | | 721 (25.44) |
| Loss of 1–8 teeth | 969 (48.94) | 1,812 (52.58) | | 1,619 (57.13) |
| Loss of 9–16 teeth | 261 (13.18) | 426 (12.36) | | 339 (11.96) |
| Loss of 17–24 teeth | 105 (5.30) | 184 (5.34) | | 99 (3.49) |
| Loss of 25–32 teeth | 82 (4.14) | 120 (3.48) | | 56 (1.98) |
| **Exercise habit** | **None**  **n=953** | **< 150 mins/week**  **n=2,917** | | **≥ 150 mins/week**  **n=4,357** |
| No tooth loss | 275 (28.86) | 801 (27.46) | | 1,103 (25.32) |
| Loss of 1–8 teeth | 463 (48.58) | 1,572 (53.89) | | 2,347 (53.87) |
| Loss of 9–16 teeth | 132 (13.85) | 325 (11.14) | | 565 (12.97) |
| Loss of 17–24 teeth | 43 (4.51) | 137 (4.70) | | 207 (4.75) |
| Loss of 25–32 teeth | 40 (4.20) | 82 (2.81) | | 135 (3.10) |

**Additional Table 4. Baseline Characteristics of Included and Excluded Participants**

|  | **Included Participants** | **Excluded Participants** | **P-value** |
| --- | --- | --- | --- |
|  | **n=8,260 (24.46%)** | **n=25,514 (75.54%)** |  |
|  | **n (% in column) / Mean ± SD** | |  |
| **Sex** |  |  |  |
| Female | 4,026 (48.74) | 13,451 (52.72) | <0.01^✝^ |
| Male | 4,234 (51.26) | 12,063 (47.28) |  |
| **Age** | 75.46 ± 6.05 | 75.07 ± 7.29 | <0.01^✝^ |
| **Marital Status** |  |  |  |
| Married | 6,723 (81.39) | 19,067 (74.73) | <0.01^✝^ |
| Unmarried | 1,536 (18.60) | 6,413 (25.14) |  |
| Missing | 1 (0.01) | 34 (0.13) |  |
| **Education Attainment** |  |  |  |
| ≥ 13 years | 2,834 (34.31) | 6,969 (27.31) | <0.01^✝^ |
| 7-12 years | 3,446 (41.72) | 10,600 (41.55) |  |
| ≤ 6 years | 1,980 (23.97) | 7,945 (31.14) |  |
| **Solitary Status** |  |  |  |
| Non-solitary | 7,818 (94.65) | 23,706 (92.91) | <0.01^✝^ |
| Solitary | 442 (5.35) | 1,808 (7.09) |  |
| **Hypertension** |  |  |  |
| Without | 4,428 (53.61) | 13,905 (54.50) | 0.16 |
| With | 3,832 (46.39) | 11,609 (45.50) |  |
| **Heart Disease** |  |  |  |
| Without | 6,951 (84.15) | 20,962 (82.16) | <0.01^✝^ |
| With | 1,309 (15.85) | 4,552 (17.84) |  |
| **Diabetes** |  |  |  |
| Without | 7,286 (88.21) | 21,913 (85.89) | <0.01^✝^ |
| With | 974 (11.79) | 3,601 (14.11) |  |

^*^ P<0.05; ^✝^ P<0.01; SD: standard deviation
